# GAS6 ameliorates advanced age-associated meiotic defects in mouse oocytes by modulating mitochondrial function

**DOI:** 10.18632/aging.203328

**Published:** 2021-07-26

**Authors:** Kyeoung-Hwa Kim, Eun-Young Kim, Kyung-Ah Lee

**Affiliations:** 1 Department of Biomedical Science, Institute of Reproductive Medicine, College of Life Science, CHA University, Seongnam-si, Gyeonggi-do 13488, Korea

**Keywords:** Gas6, aged oocyte, meiotic defect, mitochondria, fertility

## Abstract

Previously, we reported that the silencing of *growth arrest-specific gene 6* (*Gas6*) expression in oocytes impairs cytoplasmic maturation by suppressing mitophagy and inducing mitochondrial dysfunction, resulting in fertilization failure. Here, we show that oocyte aging is accompanied by an increase in meiotic defects associated with chromosome misalignment and abnormal spindle organization. Intriguingly, decreased *Gas6* mRNA and protein expression were observed in aged oocytes from older females. We further explored the effect of GAS6 on the quality and fertility of aged mouse oocytes using a GAS6 rescue analysis. After treatment with the GAS6 protein, aged oocytes matured normally to the meiosis II (MII) stage. Additionally, maternal age-related meiotic defects were reduced by GAS6 protein microinjection. Restoring GAS6 ameliorated the mitochondrial dysfunction induced by maternal aging. Ultimately, GAS6-rescued MII oocytes exhibited increased ATP levels, reduced ROS levels and elevated glutathione (GSH) levels, collectively indicating improved mitochondrial function in aged oocytes. Thus, the age-associated decrease in oocyte quality was prevented by restoring GAS6. Importantly, GAS6 protein microinjection in aged oocytes also rescued fertility. We conclude that GAS6 improves mitochondrial function to achieve sufficient cytoplasmic maturation and attenuates maternal age-related meiotic errors, thereby efficiently safeguarding oocyte quality and fertility.

## INTRODUCTION

In women, the oocyte is a reproductive cell that acquires developmental competence during growth and maturation. Oocyte maturation is an elaborate process that is uniquely regulated by an enormous number of intra- and extraovarian factors. The maturation of mammalian oocytes, involving nuclear and cytoplasmic maturation [1], prepares oocytes for successful fertilization and early embryogenesis [2]. During nuclear maturation, meiotic division involves the resumption of meiosis I (MI), condensation and redistribution of chromosomes, spindle formation, polar body extrusion and meiotic arrest in metaphase II (MII) [3].

Additionally, cytoplasmic maturation mainly involves a cytoplasmic process encompassing a wide array of metabolic and structural modifications that occur concurrently with nuclear maturation and oocyte growth [4]. Nuclear maturation is very easily identified based on the appearance of the first polar body under a microscope. However, the degree of oocyte cytoplasmic maturation is more difficult to assess beyond evaluating the fertilization ability and embryonic developmental competence of an oocyte. For normal fertilization and subsequent early embryogenesis, synchronous nuclear and cytoplasmic maturation are required in oocytes [5].

Aging exerts adverse effects on fertility. Female fertility naturally starts to decline in the early thirties and occurs more rapidly after age 37 [6]. Age-related female reproductive decline has been reported to have several causes, including oocyte diminution, disturbed hormone levels, meiotic defects, mitochondrial malfunctions and reduced oocyte quality [7]. In addition, maternal aging triggers a cascade of molecular alterations that lead to spindle assembly defects and chromosome misalignment in oocytes [8, 9]. These molecular alterations are likely to be consequences of inadequate amounts of ATP in oocytes [10]. Notably, oxidative stress is a prominent mediator associated with maternal aging that causes mitochondrial dysfunction and ATP deficiency in oocytes [11]. Mitochondria, the energy factories of oocytes, are the most abundant organelles in the ooplasm, providing ATP for transcription and translation during oocyte maturation, fertilization and early embryo development [12]. Due to the rapid elimination of sperm mitochondria after fertilization, embryonic mitochondria are derived solely from the oocyte. Thus, the quality of oocyte mitochondria determines the quality of the embryo [10]. Oocyte mitochondria have long been a popular topic of research aiming to improve oocyte competence and delay aging-related quality decline.

Fertility is an essential element of reproductive health, and infertility is recognized as a global health problem by the World Health Organization (WHO). Assisted reproductive technology (ART) has been routinely used for the treatment of infertility in humans during the last four decades [13]. Because the failure of ART is mainly attributed to low oocyte quality and increased aneuploidy, healthy oocytes are extremely important for successful ART. Despite the many advances in ART for improving fertility, oocyte aging is one of the main problems causing ART failure that is difficult to address [14]. Thus, innovative methods that increase oocyte quality are needed in ART procedures to prevent the oocyte aging process. More recently, the improvement of mitochondrial function via antioxidant therapy has been proposed as an important strategy to enhance oocyte quality [15]. Notably, melatonin and coenzyme Q10 are known antioxidants with antiaging effects on mouse oocytes by regulating mitochondrial functions and decreasing ROS levels in oocytes during maternal aging [16–18]. Many approaches for improving oocyte quality and/or rescuing fertility in older women, including supplementation with mitochondrial nutrients such as melatonin and L-carnitine, have been implemented in clinical settings [19, 20].

*Gas6* is expressed in many human tissues and regulates a number of biological processes, including immunity, proliferation, cell survival, platelet activation, and diseases [21, 22]. Moreover, GAS6 functions in a wide variety of cancers, and analysis of the tumor microenvironment suggests that GAS6 is a therapeutic target [23]. Previously, despite the higher levels of *Gas6* expression in oocytes during meiosis, *Gas6* was found not to be required for nuclear maturation. However, intriguingly, *Gas6* is involved in sufficient cytoplasmic maturation [24]. Through a subsequent series of studies, *Gas6*-silenced MII oocytes exhibited failure of pronuclear (PN) formation because of insufficient cytoplasmic maturation resulting from mitochondrial dysfunction and mitophagy suppression [25, 26]. Although GAS6 has been implicated in multiple critical biological processes in mouse oocytes, its physiological functions in oocyte quality and fertility during maternal reproductive aging have not been established. In this study, we observed decreased *Gas6* expression in oocytes with advancing age. Therefore, the objectives of the present study were to assess whether the restoration of GAS6 would overcome the meiotic defects and mitochondrial dysfunction observed in oocytes with advancing age and to determine whether maternal aging-induced subfertility would be improved by regulating oocyte mitochondrial function through GAS6 supplementation.

## RESULTS

### *Gas6* expression in mouse oocytes decreased with maternal aging

**Figure 1:**
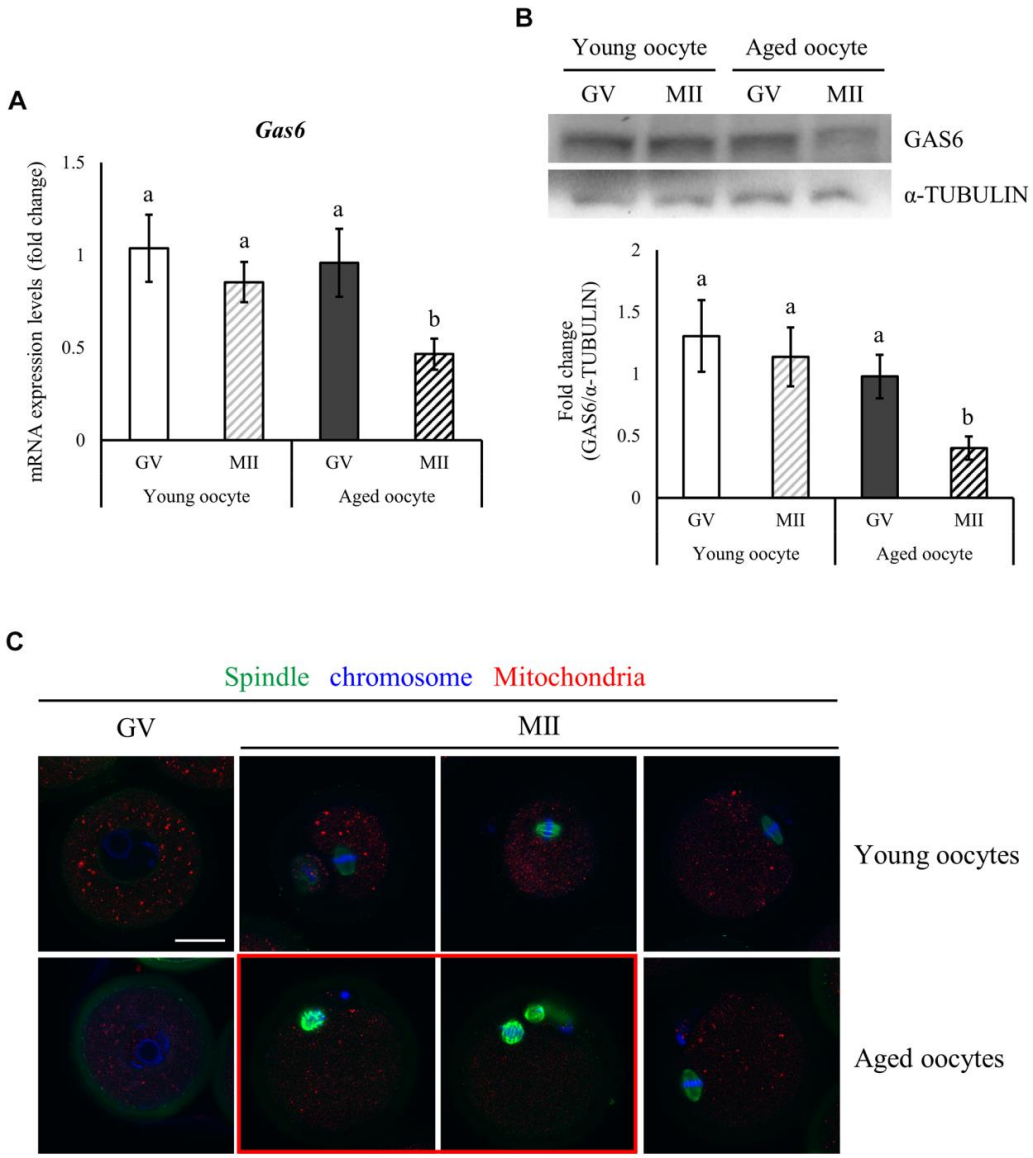
**Reduced expression of *Gas6* and increased meiotic errors in oocytes with maternal aging.** (**A**, **B**) Typical expression patterns of the *Gas6* transcripts (**A**) and protein (**B**) in GV and MII oocytes from young and aged female mice, respectively. *Gas6* expression was significantly decreased in aged oocytes. The levels of the GAS6 protein are presented in a bar graph. The data are presented as the means ± SEM. Different letters indicate significant differences at *p*<0.05. Young oocytes were obtained from 3-week-old female mice; aged oocytes were obtained from 12-month-old female mice. (**C**) Maternal aging causes spindle organization defects and chromosome misalignment during oocyte maturation. Green, spindle; blue, chromosome; red, mitochondria; red box, oocyte with abnormal spindle and chromosome alignment. The scale bars represent 25 μm.

In mouse oocytes, mRNA and protein expression levels are altered with aging. We examined *Gas6* expression in young (3 weeks) and aged (12 months) mice using qRT-PCR and Western blot analyses to determine whether age affects *Gas6* expression in the mouse oocyte. Briefly, abundant *Gas6* mRNA levels in young oocytes did not change during oocyte meiotic maturation (Figure 1A); thus, the GAS6 protein level was maintained in the young MII oocytes (Figure 1B). However, abundant *Gas6* transcript level observed in the germinal vesicle (GV) stage in aging mice was markedly reduced during meiosis, resulting in a decrease in the *Gas6* mRNA level and translational repression in aged MII oocytes (Figure 1B). Thus, the expression of *Gas6* mRNA and protein was lower in the MII oocytes of aging mice than in those of young mice (Figure 1A, 1B). Previous studies showed that the disruption of oocyte *Gas6* alone leads to fertilization failure caused by insufficient oocyte cytoplasmic maturation [24, 26]. These results revealed that inadequate *Gas6* expression in oocytes due to maternal aging led to impaired oocyte cytoplasmic maturation.

An increasing maternal age may result in a gradual decrease in oocyte quality and developmental potential. Oocyte competence is also determined by spindle morphology and chromosome alignment [27]. Spindles and chromosomes were visualized using immunofluorescence staining to observe changes in the spindle shape and chromosome configuration in oocytes with aging. Young and aged oocytes developed to the MII stage. Young MII oocytes exhibited barrel-shaped spindles with slightly pointed and well-aligned regular chromosomes along spindle equatorial plane (Figure 1C). However, some of the aged MII oocytes presented spindles with an irregular microtubule distribution and reduced length and chromosomes that did not align on the spindle equatorial plane (Figure 1C, red box). Aged MII oocytes might be unable to maintain the spindle morphology and the connection between the spindle and chromosomes.

### GAS6 alleviates advanced age-associated meiotic defects

**Figure 2:**
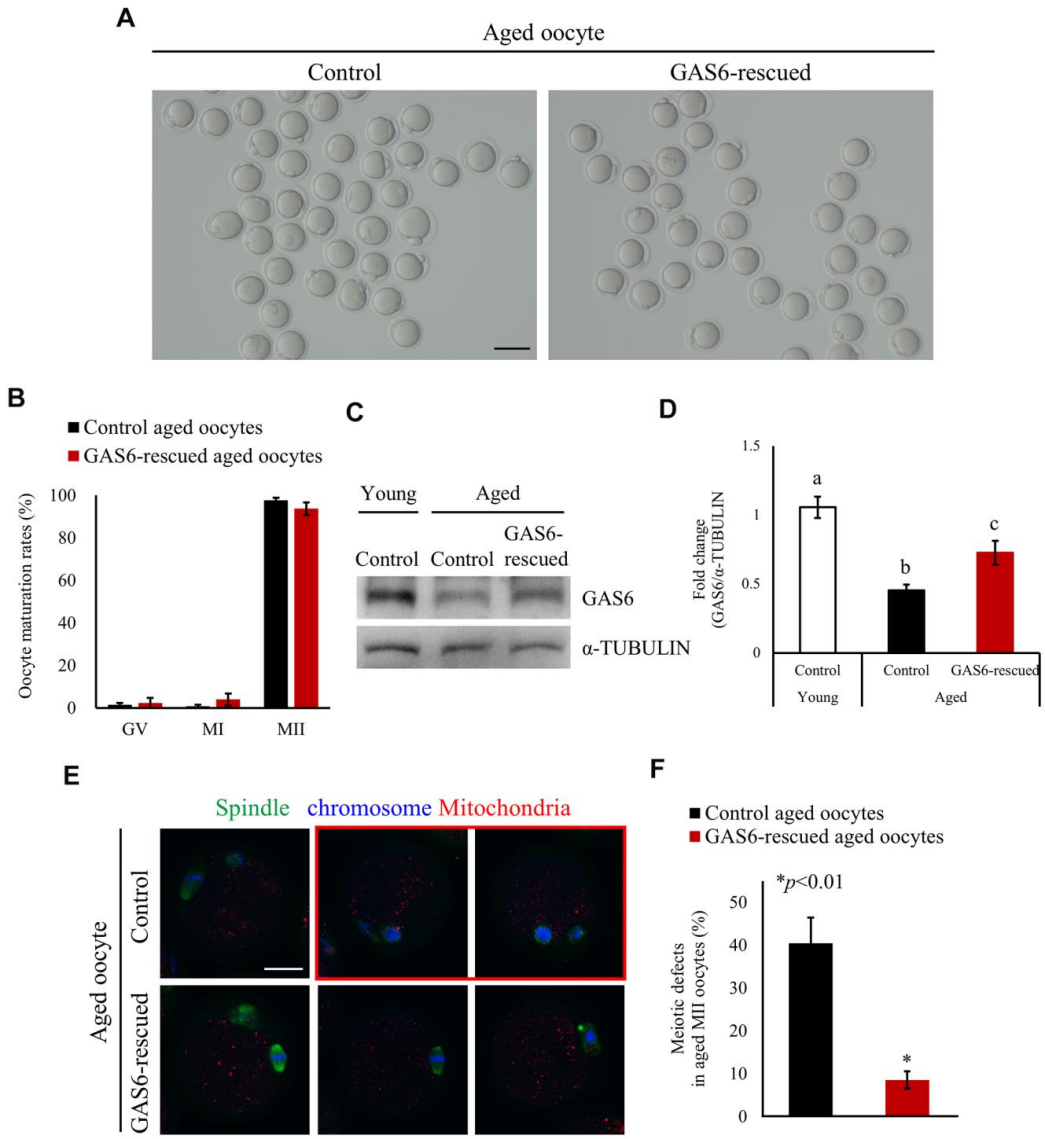
**GAS6 ameliorates age-associated meiotic defects in oocytes.** (**A**) Micrographs of aged MII oocytes treated without or with the GAS6 protein. Control, nontreated MII oocyte; GAS6-rescued, MII oocyte treated with the GAS6 protein. The scale bar represents 100 μm. (**B**) *In vitro* maturation rates of aged mouse oocytes after the microinjection of the GAS6 protein into GV-stage oocytes. After GAS6 restoration, these oocytes developed into morphologically normal MII oocytes, similar to the control groups. The data are presented as the means ± SEM. (**C**, **D**) Microinjection of the GAS6 protein led to a partial recovery of the decreased GAS6 levels induced by maternal aging. α-TUBULIN was used as a loading control. Relative expression levels of the GAS6 protein are presented in a bar graph and compared to the control young oocyte group. The data are presented as the means ± SEM. Different letters indicate significant differences at *p*<0.05. Control, nontreated oocyte; GAS6-rescued, oocyte treated with the GAS6 protein; Young, oocytes obtained from 3-week-old female mice; Aged, oocytes obtained from 12-month-old female mice. (**E**) Immunofluorescence staining for spindles and chromosomes in aged MII oocytes after injection of the GV oocytes without (control) or with the GAS6 protein (GAS6-rescued), which were then allowed to mature *in vitro*. Green, spindle; blue, chromosome; red, mitochondria; red box, oocyte with abnormal spindle and chromosome alignment. The scale bars represent 25 μm. (**F**) Proportions of control or GAS6 protein-injected MII oocytes with defects in spindle assembly and chromosome configuration.

Female fecundity is known to decline with age. In Figure 1, we noted reduced *Gas6* expression with maternal aging. We conducted rescue experiments to observe whether increasing GAS6 in aged oocytes would improve their deficient phenotypes. The GAS6 protein was injected into aged GV oocytes and meiotic progression was analyzed to address this question. As shown in Figure 2A, almost all of the GAS6-rescued aged oocytes developed to the MII stage. The maturation rate of the GAS6-rescued aged oocyte group (93.7%) was not significantly different from that of control aged oocyte group (97.6%; Figure 2B). Indeed, expression of the GAS6 protein in these MII oocytes was increased compared with that in the control aged MII oocytes (Figure 2C, 2D). Most importantly, GAS6 restoration in oocytes significantly reduced maternal age-associated meiotic defects compared with those observed in the control aged oocytes (8.6% vs. 40.5%; Figure 2E, 2F). Notably, the restoration of GAS6 in aged MII oocytes resulted in the formation of a bipolar spindle and chromosome assembly at the equatorial plane (Figure 2E). In contrast, we observed that control aged MII oocytes showed a high frequency of meiotic defects with disorganization of the spindle and chromosomal architecture (Figure 2E, red box and Figure 2F, black bar). These observations suggest that GAS6 in aged oocytes is crucial for proper organization of the spindle and normal chromosome architecture.

### GAS6 reverses maternal aging-induced mitochondrial deterioration and metabolic dysfunction in oocytes

**Figure 3:**
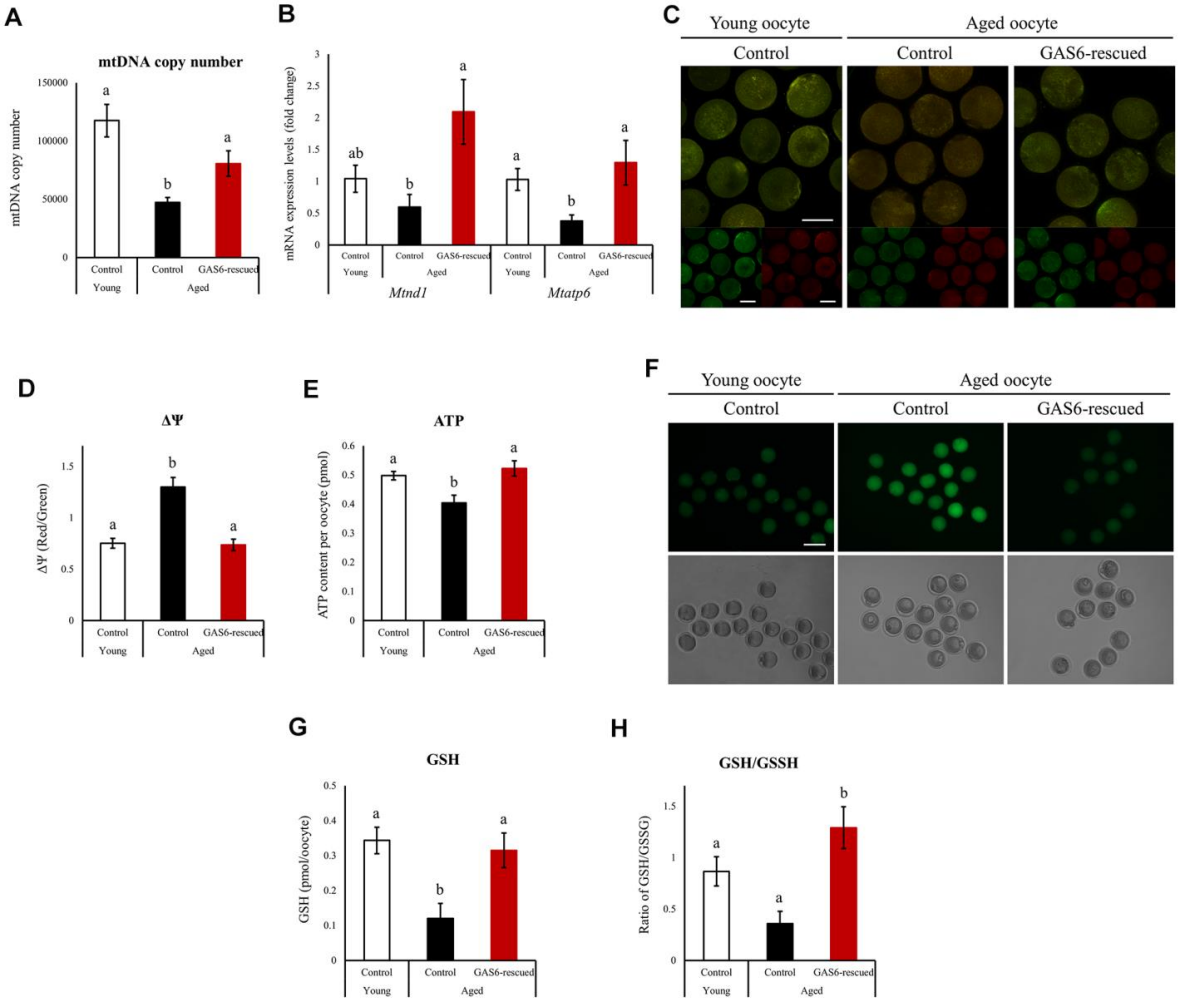
**Improvements in mitochondrial function in oocytes during aging after GAS6 supplementation.** (**A**) Measurements of the mtDNA copy numbers in aged oocytes expressing GAS6. The mtDNA copy number was higher in GAS6-rescued aged oocytes than in control aged oocytes. Different letters indicate significant differences at *p*<0.05. Control, nontreated oocyte; GAS6-rescued, oocyte treated with the GAS6 protein; Young, oocytes obtained from 3-week-old female mice; Aged, oocytes obtained from 12-month-old female mice. (**B**) Expression of *Mtnd1* and *Mtatp6* in aged MII oocytes from the control and GAS6-rescued groups. Different letters indicate significant differences at *p*<0.05. (**C**) Representative images of ΔΨm in aged MII oocytes after GAS6 supplementation. ΔΨm indicates the ratio of RITC (J-aggregate, high membrane potential) to FITC (J-monomer, low membrane potential) intensity in GAS6-rescued aged MII oocytes. Scale bars represent 50 μm. (**D**) Graphic representation of the results shown in C. The data are presented as the means ± SEM. Different letters indicate significant differences at *p*<0.001. (**E**) Effects of GAS6 restoration on ATP levels in aging oocytes. Increasing GAS6 expression resulted in elevated mitochondrial ATP production. Different letters indicate significant differences at *p*<0.05. (**F**) Treatment with GAS6 reduced ROS levels in young and aged MII oocytes. After treatment, MII oocytes were cultured in M16 medium supplemented with DCFH-DA to evaluate ROS levels. Scale bars represent 100 μm. (**G**) GAS6 treatment increased oocyte GSH levels with aging. The data are presented as the means ± SEM. Different letters indicate significant differences at *p*<0.05. (**H**) The ratio of GSH to GSSG in GAS6-rescued aged MII oocytes was calculated. The GSH/GSSG ratio is a biochemical marker of oxidative stress. The GSH/GSSG ratio was increased in aged MII oocytes after the restoration of GAS6. Thus, GAS6 reduces the oxidative stress caused by maternal aging. Different letters indicate significant differences at *p*<0.05.

In mice and humans, mtDNA copy numbers and mitochondrial function are closely related to oocyte quality [15]. As previously reported, mtDNA copy numbers are reduced in oocytes from aged mice [28]. Because a reduction in *Gas6* expression resulted in a decreased mtDNA content in young MII oocytes, we examined whether the restoration of GAS6 would rescue the mtDNA copy number in oocytes with maternal aging. We therefore examined the mtDNA content in aged oocytes with or without GAS6 treatment using qRT-PCR analysis. Young control MII oocytes contained an average of 117,579 ± 13,922 mtDNA copies per oocyte (Figure 3A). However, control aged MII oocytes contained an average of 48,650 ± 4,840 mtDNA molecules (Figure 3A). The mtDNA copy number decreased by approximately 50% with oocyte aging. Importantly, GAS6-rescued aged oocytes contained an average of 74,040 ± 7,750 mtDNA molecules (Figure 3A). As shown in Figure 3B, the transcript levels of the mtDNA-encoded genes *Mtnd1* and *Mtapt6* were reduced in aged MII oocytes. After GAS6 protein microinjection in aged MII oocytes, however, the *Mtnd1* and *Mtapt6* transcripts were significantly increased to levels similar to those observed in young control group (Figure 3B). Mitochondrial function is an important prerequisite for oocyte cytoplasmic maturation and subsequent fertilization and embryo development [10, 15, 16]. Accordingly, we used a mitochondrial tracker to examine active mitochondria in aged oocytes after restoring GAS6. Red and green signals indicated mitochondria with a high ΔΨm and low ΔΨm, respectively (Figure 3C). As shown in Figure 3C, 3D, the red/green intensity ratio increased with advanced maternal age. In contrast, ΔΨm was apparently reduced in GAS6-rescued aged oocytes, similar to the young control group (Figure 3C, 3D). Aged oocytes display numerous abnormalities, including a reduction in the ATP content. The decrease in ATP levels exerts a deleterious effect on fertilization and early embryogenesis. We also evaluated mitochondrial ATP production in aged oocytes treated with GAS6. Compared with young control MII oocytes, aging of oocytes resulted in significantly decreased intracellular ATP levels (Figure 3E). However, restoration of GAS6 in aged oocytes resulted in a marked increase in intracellular ATP (Figure 3E). Mitochondrial dysfunction is one of the major factors that induces an increase in ROS levels; therefore, we assessed changes in ROS levels associated with maternal aging and GAS6 treatment in oocytes. As shown in Figure 3F, compared to young control MII oocytes, ROS levels were markedly increased in control aged MII oocytes, while intracellular ROS levels were considerably reduced after GAS6 restoration in aged MII oocytes. Intracellular glutathione (GSH) contributes to oxidative stress resistance and protects oocytes from oxidative damage. Indeed, GSH levels reflect oocyte competence and quality. However, intracellular GSH levels in oocytes gradually decrease with increasing maternal age [29]. We analyzed intracellular GSH levels in MII oocytes treated with or without the GAS6 protein to examine the effect of GAS6 on GSH synthesis during the aging process. As expected, GSH levels and the GSH/GSSG ratio were decreased in the aged oocytes (Figure 3G, 3H). However, both GSH contents and GSH/GSSG ratio in aged MII oocytes were markedly increased after GAS6 restoration, similar to the young control group (Figure 3G, 3H). Altogether, these observations indicated that GAS6 prevented maternal aging-induced mitochondrial dysfunction in oocytes and increased the quality of aged oocytes by modulating metabolism.

### GAS6 improves the maternal aging-induced decrease in fertility and promotes the fertilization ability of young oocytes

**Figure 4:**
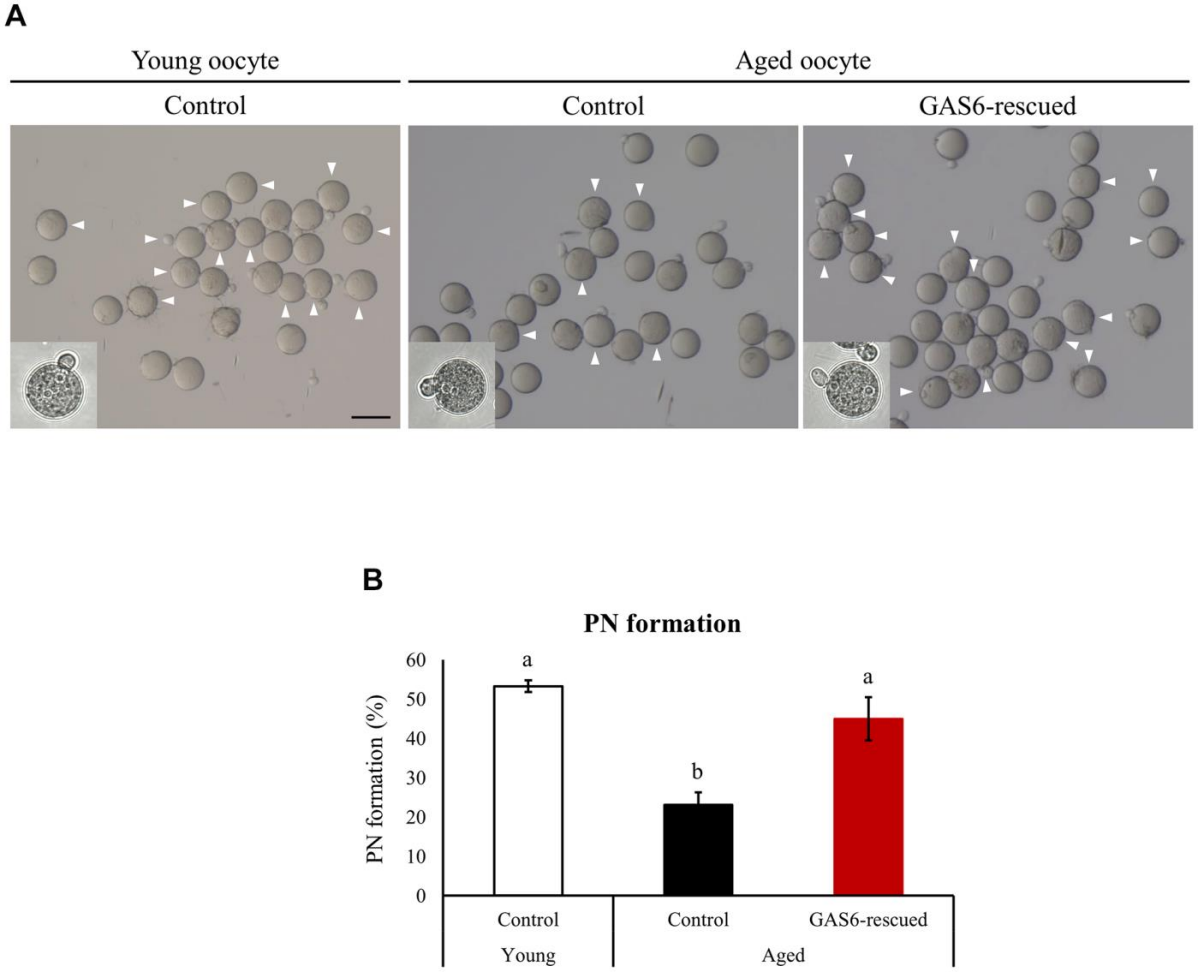
**GAS6 restores the *Gas6* silencing- and/or maternal age-induced decline in fertility.** (**A**) Microphotographs of PN embryos after *in vitro* fertilization. Aged oocytes were treated without (control) or with the GAS6 protein (GAS6-rescued) and then fertilized after *in vitro* culture. White triangles indicate PN formation. Scale bars represent 100 μm. (**B**) Percentage of oocytes showing PN formation after *in vitro* fertilization. Experiments were repeated at least three times, and data are presented as the means ± SEM. Different letters indicate significant differences at *p*<0.05.

**Figure 5:**
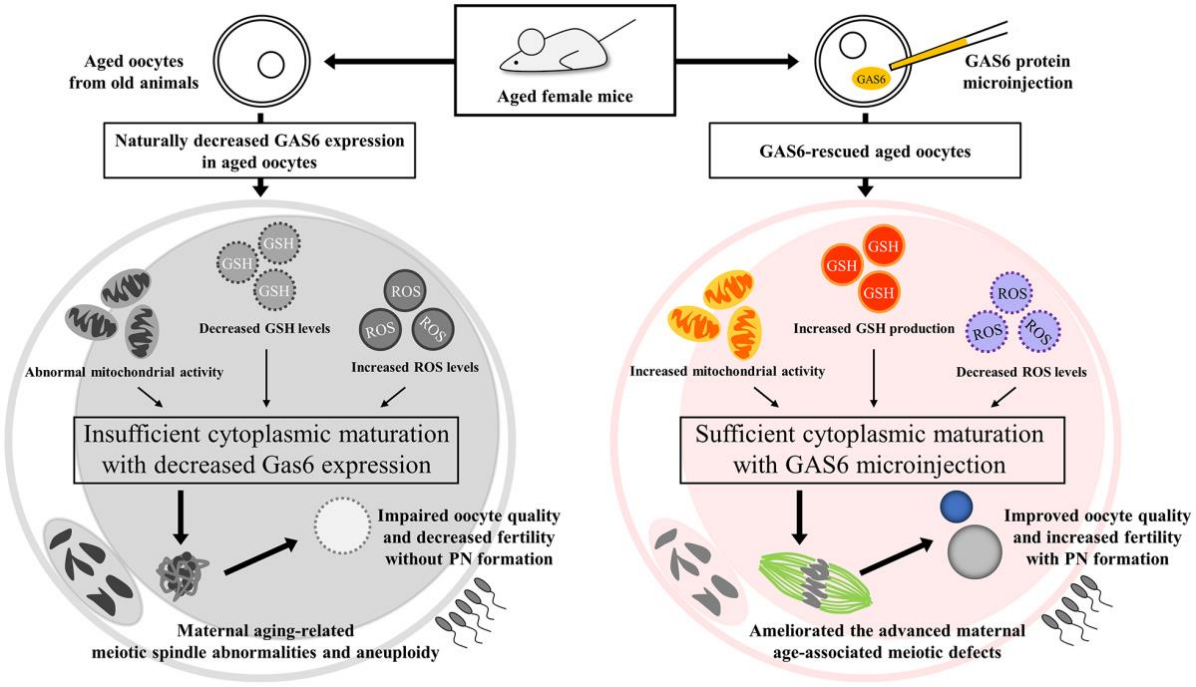
**GAS6 attenuates the decrease in the quality of aged oocytes.** Aged oocytes showed increased mitochondrial deterioration, elevated oxidative stress and insufficient cytoplasmic maturation due to decreased *Gas6* expression, resulting in meiotic defects and consequential PN formation failure. Moreover, GAS6-rescued aged oocytes collectively exhibited metabolic alterations, indicating better-quality mitochondrial functions. Therefore, the age-associated decreases in oocyte quality and fertility were prevented by restoring GAS6 levels.

*Gas6*-silenced oocytes failed to undergo normal fertilization [24]. Because *Gas6* expression decreases in oocytes with age, contributing to a decrease in the oocyte quality, we sought to determine whether the fertilization ability would be restored by the GAS6 treatment. We increased GAS6 levels by injecting the GAS6 protein to address these questions. Control aged oocytes exhibited a markedly lower fertilization rate than young control oocytes (23.1% vs. 53.3%; Figure 4A, 4B). As expected, in the GAS6-rescued aged oocyte group, the rate of PN formation was effectively increased compared with that in control aged oocytes (Figure 4B). After GAS6 rescue, the percentage of PN formation was approximately double that in the control aged oocytes (23.1% vs. 45.0%; Figure 4B). Remarkably, the percentage of PN formation in the GAS6-rescued aged oocytes was not substantially different than that in the control young oocytes (53.3% vs. 45.0%; Figure 4B). Collectively, these findings strongly suggest that GAS6 protects oocytes from the deterioration of oocyte competence during reproductive aging and improves fertilization ability (Figure 5).

## DISCUSSION

We identified an important role for GAS6 in controlling meiosis via the regulation of mitochondrial function that is important for oocyte quality under conditions of maternal reproductive aging. In mice, decrease in *Gas6* expression was detected in MII oocytes from older animals. We also observed that meiotic defects appear to be a major factor that contributes to chromosome misalignment and spindle abnormalities in aging. However, GAS6 restoration in oocytes rescued maternal age-associated meiotic defects, and thus it is directly responsible for oocyte competence. The restoration of GAS6 in aged mouse oocytes restores mitochondrial function and metabolism; the associated changes include increased mtDNA contents and mtDNA-encoded gene transcription, ΔΨm alterations, increased intracellular ATP levels, reduced ROS levels, and elevated GSH levels. Furthermore, GAS6 restoration in oocytes enhances the fertilization capacity of aged oocytes. Thus, studies addressing how maternal cytoplasmic factors may contribute to the developmental competence of oocytes with aging are also critical. We propose that GAS6 improves oocyte quality and competence with advanced maternal age to increase fertilization and early embryo developmental rates.

The spindle is a major cytoplasmic structure involved in the accurate segregation and alignment of chromosomes in both meiosis and mitosis. In oocytes, meiotic spindle abnormalities and aneuploidy become much more prevalent with aging. The increase in meiotic defects with reproductive aging leads to increased rates of infertility, miscarriage and fetal trisomy [30]. In rodents, GAS6 levels in tissues and serum decrease with aging, which may contribute to the aging process [31]. Here, we report that the expression of *Gas6* also decreases with aging in mouse oocytes. Our current study confirms that GAS6 reverses the disruption of meiotic spindle assembly and chromosome segregation that occurs during aging. In cancer cells, GAS6-activated AXL induces multiple actin-dependent cytoskeletal rearrangements that jointly contribute to invasion. In oocytes, actin closely interacts with microtubules during meiosis. The actin-microtubule interaction is essential for formation of the bipolar spindle, emission of first polar body, and metaphase II arrest [32]. Inadequate *Gas6* expression in oocytes due to maternal aging may lead to meiotic defects via an impaired actin-microtubule interaction. Hence, mouse oocytes appear to be exquisitely sensitive to decreased GAS6 levels during meiosis.

Previous studies involving mouse and human oocytes have shown that mtDNA levels are inversely correlated with maternal age [10]. In other words, oocytes with a higher mtDNA copy number show greater potential for successful fertilization and early embryogenesis [33]. Furthermore, in human and mouse MII oocytes, critical thresholds of approximately 100,000 mtDNA copies and approximately 50,000 mtDNA copies, respectively, must be met for fertilization and embryonic development, suggesting that mtDNA copy number may be an indicator of oocyte competence [34, 35]. In this study, MII oocytes in which *Gas6* was downregulated exhibited a reduction in the mtDNA content with maternal aging. The decrease in mtDNA levels caused by reduced *Gas6* expression in aged MII oocytes below the threshold led to the inhibition of PN formation following *in vitro* fertilization. However, we observed a marked increase in mtDNA content in aged MII oocytes after GAS6 restoration. Thus, *Gas6* downregulation in oocytes is involved in adverse effects on mtDNA levels and contributes to the deterioration of oocyte quality. However, the results do not support the direct association of GAS6 with the mtDNA content in oocytes. The mechanism by which GAS6 regulates mtDNA levels in mouse oocytes should be studied in the near future.

In parallel, we assessed the effect of GAS6 on the age-dependent mitochondrial dysfunction of mouse oocytes. Mitochondria in oocytes synthesize ATP and are thus critical for providing cellular energy to maintain the developmental competence of oocytes. An appropriate ΔΨm is required to maintain intracellular ATP pools. However, an excessively high ΔΨm favors the deleterious accumulation of ROS [36]. Notably, an appropriate intracellular redox state and mitochondrial function in oocytes are essential for proper formation and maintenance of the spindle apparatus. In addition, chromosome condensation is dependent on ATP levels in oocytes. Based on these observations, insufficient ATP levels and elevated ROS levels in oocytes during meiosis are linked to meiotic defects [27, 37]. Thus, our data revealed that mitochondria in aged oocytes maintained a higher ΔΨm but did not preserve ATP production, leading to a reduction in mitochondrial ATP levels. In aged oocytes, the induction of ROS production reduces GSH levels and increases meiotic defects. In contrast, in aged oocytes with GAS6 restoration, proper mitochondrial ATP production resulting from an appropriate ΔΨm leads to a reduction in oxidative stress and thereby promotes GSH production. Importantly, we observed a lower frequency of maternal age-associated meiotic defects in oocytes after GAS6 restoration, suggesting that the increase in ATP levels in GAS6-restored aged oocytes is essential for adequate energy metabolism for the proper formation and function of spindle structures during meiosis. Hence, we concluded that GAS6 in oocytes is a pivotal regulator of redox homeostasis, GSH production and the meiotic apparatus through its effects on mitochondrial function.

We are first to report that restoration of GAS6 in aged oocytes alleviates age-associated oocyte aging and improves impaired fertility by promoting sufficient oocyte cytoplasmic maturation. However, our study may have some limitations. First, only mouse oocytes were included in this study. To date, direct evidence of any effects of GAS6 on human oocytes is unavailable. Thus, further studies are necessary to confirm how GAS6 might affect human oocyte competence with aging. Second, aged oocyte competence after GAS6 treatment was assessed only until fertilization, and thus the study cannot reveal its effects on early embryogenesis. Third, the optimal dose of the GAS6 protein to restore age-related fertility decline should be evaluated in the future. In addition, safety issue should be addressed before applying it as an ART.

The decreases in oocyte quality and ovarian reserves that occur with advanced maternal age are currently the major challenges in the field of ART. Infertility therapy is associated with oxidative stress, which has the potential to negatively affect oocyte quality. In humans and mice, numerous studies have documented the effective role of antioxidants in preventing maternal age-related oocyte defects [16, 20, 38, 39]. We also found that GAS6 ameliorated maternal age-related meiotic and energy metabolism defects, resulting in the restoration of adequate levels of ROS in oocytes in particular. However, we did not verify this finding in human oocytes. Thus, this issue needs to be addressed in further research in humans. Importantly, a well-formed spindle architecture through well-balanced and well-timed energetic metabolism during meiosis is an essential for the acquisition of oocyte developmental competence [10, 39, 40]. Consistent with this concept, *Gas6* is a new therapeutic target that may exert positive effects on oocyte quality in women of advanced age. Therefore, we conclude that GAS6 treatment in oocytes may be proposed as an adjunct therapy in the treatment of the infertility and/or subfertility of aged oocytes in women of advanced maternal age.

## MATERIALS AND METHODS

### Animals

Female imprinting control region (ICR; young, 3 weeks; aged, 12 months) mice, which were exclusively provided by Koatech (Pyeoungtack, Korea) and Envigo (Horst, Netherlands). We used 200 female mice at 12 months of age for the GAS6 rescue experiments. All procedures were performed in accordance with institutional guidelines and had been approved by the Institutional Animal Care and Use Committee of CHA University.

### Reagents and antibodies

Unless otherwise stated, chemicals and reagents were purchased from Sigma-Aldrich (St. Louis, MO, USA). Goat anti-GAS6 (sc-1936) and mouse anti-α-TUBULIN (sc-8035) were obtained from Santa Cruz Biotechnology (Dallas, TX, USA).

### Isolation of oocytes by ovarian stimulation

Oocyte isolation was performed as previously reported [25]. The ICR mice strain was used for all animal experiments. Female mice were sacrificed 46 hours after the intraperitoneal injection of PMSG. To prevent meiotic progression during oocyte collection and microinjection, we used M2 medium containing 0.2 mM IBMX. The isolated ovaries were dissociated in M2 medium containing IBMX. After the cumulus-oocyte complexes had been collected, cumulus cells were mechanically detached from the oocytes, and fully grown denuded GV oocytes were collected for microinjection experiments. The cumulus-oocyte complexes were cultured to obtain MII oocytes using a previously described protocol [25]. After washing with PBS-PVA, 20 or 50 oocytes from each group were transferred into a microcentrifuge tube and immediately snap frozen. The oocytes were stored at -80° C until the experiments were performed.

### Oocyte microinjection

Recombinant mouse GAS6 protein was purchased from R&D Systems (8310-GS; Minneapolis, MN, USA). The GAS6 protein was diluted to final concentration of 5 ng/μl. Aged GV oocytes were randomly grouped and microinjected with the GAS6 protein using a microinjector (Transjector; Eppendorf, Hamburg, Germany) for the GAS6 rescue assay. After protein microinjection, the oocytes were thoroughly washed and cultured in M16 medium supplemented with IBMX for 4 to 6 hours with 5% CO_2_ at 37° C. The GAS6-rescued aged oocytes were washed and then cultured for 16 hours in M16 medium. To determine the oocyte stage, morphological changes were documented as previously described [24].

### RNA isolation from oocytes and qRT-PCR

To detect *Gas6* transcript expression during the aging process, mRNA was isolated from 20 GV-stage oocytes using the Dynabeads mRNA DIRECT kit (Ambion, Austin, TX, USA). The mRNA isolated from each sample (20 oocytes) was reverse transcribed for cDNA synthesis using M-MLV RT (Promega) with oligo (dT) primer. The synthesized cDNA was assessed by qRT-PCR. All assays used cDNA from a single oocyte for each PCR sample. The sequences of the gene-specific primers are listed in Supplementary Table 1. The expression of *Gas6* and mitochondrially encoded transcripts was measured using iQ SYBR Green Supermix (Bio-Rad, Hercules, CA, USA) with a CFX96 Touch^TM^ Real-Time PCR Detection System (Bio-Rad), and then results were assessed using CFX Maestro software (Bio-Rad). Melting curve analysis was used to determine the specificity of the amplified products. *H1foo* was used to normalize the expression of each gene. Relative changes in gene expression were quantified using the comparative C_T_ method.

### Western blotting

In samples containing an equal number of oocytes, protein expression was evaluated by Western blotting using a standard protocol. The bands were viewed using the ChemiDoc XRD+ system (Bio-Rad). α-TUBULIN served as a control. The relative intensity of each band was evaluated using Image Lab software (Bio-Rad).

### Mitochondrial staining with mitotracker and JC-1

Mitochondria were examined using MitoTracker Orange CMTMRos (Molecular Probes, Eugene, OR, USA). Briefly, oocytes were cultured in M16 medium containing 300 nM MitoTracker for 30 minutes with 5% CO_2_ at 37° C. After washing with PBS-PVA, the oocytes were fixed in 3.7% paraformaldehyde for 1 hour and permeabilized in PBS-PVA-BSA containing Triton X-100 for 30 minutes. After blocking, the oocytes were stained overnight at 4° C with an anti-α-TUBULIN antibody and then incubated in PBS-PVA-BSA containing Alexa Fluor 488-conjugated secondary antibody. After washing, the DNA was stained with DAPI, and the oocytes were mounted on glass sides. The mitochondrial distribution was observed with a Leica confocal microscope (Wetzlar, Germany). At least 30 oocytes per group were assessed in three different replicates.

To evaluate the change in ΔΨm in the oocytes, oocytes were stained with MitoProbe JC-1 (Thermo Fisher Scientific, Waltham, MA, USA). Oocytes were cultured in M16 medium supplemented with JC-1 at 1 μg/mL for 30 minutes and then washed with PBS-PVA. The oocytes were mounted and immediately imaged in the red and green fluorescence channels under a confocal microscope. For quantitative analysis, LAS AF Lite software (Leica) was used to measure the signal intensities, and ΔΨm was calculated as the ratio of the red/green signal.

### Intracellular ATP levels in oocytes

Based on the ATP-dependent luciferin-luciferase reaction, intracellular ATP levels (10-20 oocytes) were examined using a Bioluminescent Somatic Cell Assay Kit (FL-ASC), as we described in a previous report [37]. Serial dilutions of an ATP standard (range of 0-10 pmol) and negative controls were prepared for each assay. The relative signal intensity of each group was assessed using a luminometer (Centro XS3 LB 960; Berthold Technologies, Bad Wildbad, Germany). A standard curve was generated using linear regression, and the intracellular ATP content per oocyte was calculated from the standard curve.

### Reduced GSH (GSH), oxidized GSH (GSSH) and ROS levels in oocytes

The levels of GSH and GSSH in the oocytes were measured using an EnzyChrom™ GSH and GSSG Assay Kit (EGTT-100; Bioassay Systems, Hayward, CA, USA). Oocytes were vortexed in cold buffer with or without a scavenger. After centrifugation, the oocyte lysates were deproteinated using metaphosphoric acid. A detection reagent containing assay buffer, NADPH, DTNB and glutathione reductase was added to deproteinate the oocyte samples. Upon the reaction of DTNB and GSH, the sample turned yellow. Finally, the optical density was measured at 412 nm using an Epoch microplate spectrophotometer. A standard curve was generated, and the GSH and GSSH levels per oocyte were calculated.

To determine ROS levels in the oocytes, intracellular ROS production was evaluated using an Oxiselect ROS assay kit (STA 342; Cell Biolabs, San Diego, CA, USA). Oocytes were cultured in M16 medium supplemented with the ROS indicator 2’,7’-dichlorodihydrofluorescein diacetate (DCFH-DA) for 1 hour at 37° C in the dark. After washing with M16 medium, the oocytes were transferred to a 96-well dish and immediately visualized using fluorescence microscopy.

### mtDNA content in the oocytes

Mitochondrial DNA was extracted from 10 MII oocytes subjected to GAS6 rescue experiments using a mitochondrial DNA isolation kit (Abcam). The mitochondrial DNA content in the oocytes was evaluated by qRT-PCR as we reported previously [41]. qRT-PCR analysis used mtDNA at an amount equivalent to a single oocyte per PCR sample. The sequences of the primers specific to *Mtnd1* are listed in Supplementary Table 1. A standard curve was generated from tenfold dilutions of purified plasmid DNA for each assay. The mtDNA copy number per single oocyte was determined from the standard curve. Each sample was analyzed in triplicate.

### *In vitro* fertilization (IVF)

For IVF experiments, M16 medium (with or without BSA) and mineral oil were prewarmed and equilibrated prior to use. Male ICR mice at 10 weeks of age were sacrificed. Sperm were obtained from the caudal epididymis (Koatech) and underwent capacitation by incubation in a 100-μl droplet of M16 medium supplemented with BSA for 1 hour with 5% CO_2_ at 37° C. To remove the zona pellucida, the oocytes were carefully and rapidly treated in acid Tyrode’s solution (pH 2.5). Zona pellucida-free MII oocytes (10-15 oocytes) were placed in a 200-μl droplet of M16 medium and incubated for 30 minutes with 5% CO_2_ at 37° C. After capacitation, 10 μl of 2.5×10^4^ sperm/ml was added to a droplet containing zona pellucida-free MII oocytes and subsequently cultured for 4 hours. To remove the sperm, the oocytes were washed extensively with M16 medium and then transferred to prewarmed plain M16 medium. After 3-4 hours, PN formation was examined under a microscope.

### Data analysis and statistics

Each experiment was repeated independently at least three times unless indicated otherwise. The results are reported as the mean ± standard error of the mean (SEM). Statistical analyses between the control aged oocyte group and GAS6-rescued aged oocyte group were performed using Student’s *t*-test. In addition, comparisons of multiple groups were carried out with one-way ANOVA. Differences for which *p*<0.05, as determined by statistical analysis, were deemed to be significant.

## Supplementary Material

Supplementary Table 1
