# Supplementary material for: GAS6 ameliorates advanced age-associated meiotic defects in mouse oocytes by modulating mitochondrial function
Source: Aging (Albany NY). 2021 Jul 26;13(14):18018–32. doi: 10.18632/aging.203328 (PMC8351714; doi:10.18632/aging.203328)
Supplement: Supplementary Table 1 [file aging-13-203328-s001.pdf]

SUPPLEMENTARY TABLE

Supplementary Table 1. Primer sequences and RT-PCR conditions.

Gene symbol	Gene name	Accession number	Primer sequences[a]
Gas6	Growth arrest-specific 6	NM_019521.2	For-AAAGGGCCAGAGTGAAGTGA<br>Rev-TTTTCCCGTTTACCTCCAGA
Mtatp6	Mitochondrially encoded ATP synthase membrane subunit 6	NC_005089.1	For-ACAGGCTTCCGACACAAACT<br>Rev-GTAGCTGTTGGTGGGCTAAT
Mtnd1	Mitochondrially encoded NADH dehydrogenase 6	NC_005089.1	For-TCGACCTGACAGAAGGAGAA<br>Rev-GATGCTCGGATCCATAGGAA
H1foo	H1 histone family, member O, oocyte-specific	NM_138311	For-GCGAAACCGAAAGAGGTCAGAA<br>Rev-TGGAGGAGGTCTTGGGAAGTAA
Mtnd1	Mitochondrially Encoded NADH:ubiquinone oxidoreductase core subunit 1	NC_005089.1	For-CAATACGCCCTTTAACAACC<br>Rev-TTTGGAGTTTGAGGCTCATC

[a]For, Forward; Rev, Reverse.
